# Characterization of aroma profiles of Tanyang Congou black tea with flowery-fruity flavor: Insights from sensory evaluation and HS-SPME-GC-O-MS

**DOI:** 10.1016/j.fochx.2025.102595

**Published:** 2025-05-28

**Authors:** Di Zhou, Xin-yu Liu, Miao-qin Xie, Hao-jie Xu, Huai-hui Yi, Da-xiang Li, Ru-yan Hou, Hui-mei Cai, Xiao-chun Wan, Daniel Granato, Chuan-yi Peng

**Affiliations:** a State Key Laboratory of Tea Plant Germplasm Innovation and Resource Utilization, Anhui Agriculture University, Hefei 230036, Anhui, People's Republic of China; b Anhui Province Key Lab of Analysis and Detection for Food Safety, Hefei 230022, Anhui, People's Republic of China; c Wuyishan Vocational College, Wuyishan 354300, Fujian, People's Republic of China; d Department of Biological Sciences, Faculty of Science and Engineering, University of Limerick, V94 T9PX, Limerick, Ireland

**Keywords:** Electronic nose, Flower and fruit aroma, Key aroma compounds, GC-O-MS, Aroma extract dilution analysis, Chemometrics

## Abstract

Tanyang Congou black tea, renowned for its distinctive floral and fruity aroma, is meticulously produced using the shaking technique. However, the specific aroma profile and the key odor-active compounds responsible for this characteristic fragrance have not been fully elucidated. This study integrated sensory evaluation with molecular sensory science approaches to identify and characterize the principal odorants contributing to the tea's aroma. Sensory analysis confirmed that the prepared black tea exhibited typical high-quality attributes, with a prominent floral and fruity aroma markedly reduced in lower-grade samples. A total of 70 volatile compounds were detected, among which 29 key aroma-active compounds were identified across all three quality grades using aroma extract dilution analysis (AEDA) and gas chromatography-olfactometry-mass spectrometry (GC-O-MS). Of these, 16 volatiles exhibited high flavor dilution (FD) factors (≥8), and 11 compounds showed relative odor activity values (ROAV) greater than 1. Notably, seven compounds—(*E*)-*β*-ionone, (E)-nerolidol, geraniol, citral, linalool, hexanal, and phenylacetaldehyde—were identified as the primary contributors to the characteristic floral-fruity aroma of Tanyang Congou black tea. These findings provide comprehensive insight into the aroma profile of Tanyang Congou black tea, offering a scientific basis for quality assessment and targeted aroma modulation in tea production.

## Highlights

• 29 key aroma compounds were identified across all three quality grades.
• 16 aroma volatiles demonstrated higher FD values above 8.
• 11 compounds with ROAVs greater than 1 were calculated.
• 7 compounds largely contributed to the floral-fruity aroma of Tanyang Congou black tea.

## 1 Introduction

Tea is a widely consumed beverage known for its unique flavor and numerous health benefits (Peng et al., 2022; Ren et al., 2023). Based on their respective manufacturing techniques, dried leaves from the evergreen tea tree are classified into several types: green tea, black tea, oolong tea, white tea, yellow tea, and brick tea (Peng et al., 2022). Of these, the typical manufacturing stages of black tea involve withering, rolling, fermentation, and drying (Chen et al., 2022), classifying it as a fully oxidized tea (Dong et al., 2024). Due to its unique, pleasant flavor and various health benefits, black tea has gained significant popularity among consumers, accounting for 75 % of global tea consumption (Chen et al., 2022; Ouyang et al., 2024).

Chinese black tea is renowned for its extraordinary aroma characteristics, distinguished by a ruby-red infusion and leaves, often accompanied by sweet, honey, or floral aromas (Dong et al., 2024). The aroma profile of black tea varies significantly by region and is influenced by factors such as tea cultivars, climate, soil composition, and manufacturing methods (Su et al., 2022; Yao et al., 2023). For instance, Keemun black tea is noted for its distinctive “Keemun aroma”, featuring floral and honey notes (Su et al., 2022), while Darjeeling black tea is recognized for its muscatel scent (Kang et al., 2019). Xinyang black tea has a honey-sugar-like aroma (Yao et al., 2023), and Hunan black tea is celebrated for its floral-honeyed fragrance (Yin et al., 2023). Aroma is a crucial factor in assessing the quality of black tea, significantly influencing consumer preferences and acceptance. High-quality black tea with floral and fruity aromas is increasingly gaining popularity. Various strategies have been explored to enhance the aroma of black tea through innovations in manufacturing, including rotation (Wang et al., 2024), red-light treatment during withering (Li et al., 2022), and the introduction of urea oxidase after crushing (Qiao et al., 2023).

Previous studies have elucidated that rotation, also known as bruising and withering, is a distinctive manufacturing technique used in the production of oolong tea. This process significantly influences the development of its floral and fruity aromas during manufacturing (Hu et al., 2018; Wang et al., 2023a). During the bruising and withering process, the tea leaves undergo ongoing mechanical damage, which encourages the accumulation of volatile aroma compounds. This transformation is essential in developing the distinctive aroma characteristic of oolong tea (Ye et al., 2023). Innovations in tea manufacturing continue to emerge, aiming to enhance flavor quality. One notable development involves integrating the shaking process used in oolong tea into the traditional manufacturing methods of white, black, and green teas to improve aroma quality (Xue et al., 2022; Wang et al., 2023a; Wang et al., 2023b; Yin et al., 2023; Wang et al., 2024). Tanyang Congou black tea, with its flowery-fruity flavor, is meticulously crafted using this innovative process (DB35/T 632., 2022). However, the aroma profile and key aroma compounds of Tanyang Congou black tea with flowery-fruity flavor remain inadequately understood.

Molecular sensory science and technology can dissect the aroma components of tea, primarily encompassing sensory evaluation and analytical detection (Zhai et al., 2022). Sensory evaluation techniques include quantitative descriptive analysis (QDA), aroma extract dilution analysis (AEDA), odor activity values (OAV), aroma addition and omission experiments, *etc.* (Feng et al., 2019; Zhai et al., 2022). Analytical detection techniques primarily include gas chromatography–mass spectrometry (GC–MS), gas chromatography-olfactometry (GC-O), internal standard semi-quantitative method, standard addition quantitative method, and stable isotope dilution analysis (SIDA), among others (Zhai et al., 2022). The study used *Camellia sinensis vs.* Jin Mudan tea variety as raw materials to prepare different Tanyang Congou black tea grades with flowery-fruity flavors in accordance with the Provincial Standards (DB35/T 632., 2022). Molecular sensory science analysis techniques were employed to construct the aroma profile, identify key aroma compounds responsible for the floral and fruity notes, and analyze the possible formation mechanism of this characteristic aroma during manufacturing. The aim was to establish a foundation for aroma quality control of Tanyang Congou black tea, characterized by a flowery-fruity flavor.

## 2 Materials and methods

### 2.1 Reagents and materials

Chromatography-grade *n*-alkanes (C7-C40) were purchased from Shanghai Aladdin Bio-Chem Technology Co., Ltd. Sodium chloride was obtained from the China National Pharmaceutical Group, and deionized water (18.2 MΩ cm) was prepared using a Milli-Q Gradient system (Billerica, MA, USA).

### 2.2 Preparation of Tanyang congou black tea with flowery-fruity flavor

**Fig. 1:**
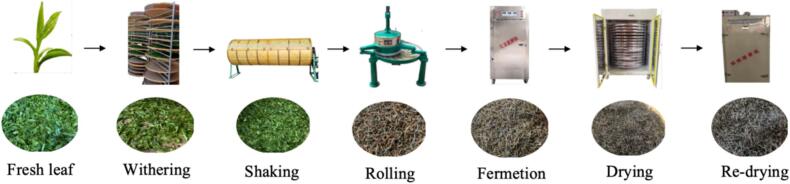
The manufacturing of Tanyang Congou black tea with flowery-fruity flavor.

The fresh tea leaves were plucked from the *Camellia sinensis* (L.) O. Kuntze *vs* Jin Mudan variety tea garden in Xiaoyang Town, Fu'an City, Fujian Province. According to the grading of fresh tea leaves for Tanyang Congou black tea (floral and fruity type) specified in DB35/T 632–2022 “Technical Specifications for Processing Tanyang Congou Black Tea,” fresh leaves of first grade (FG), second grade (SG), and third grade (TG) were collected, respectively. The fresh leaves were processed through a series of steps: withering, light shaking, rolling, fermentation, drying, and re-drying. Samples were frozen and stored in an ultra-low temperature freezer at −80 °C. The collected samples were dried in a vacuum freeze dryer at −50 °C for 72 h. After drying, samples were ground into a powder using a pre-frozen mortar and pestle, sieved through a 40-mesh screen, and stored at −22 °C for future use. The first-grade Tanyang Congou black tea, characterized by a flowery-fruity flavor (Fig. 1), was selected to investigate the mechanisms of aroma formation. Each grade of tea sample and manufacturing sample were prepared in triplicate.

### 2.3 Analytical method

#### 2.3.1 Sensory evaluation

Ethics committee approval was not required for human sensory evaluations at Anhui Agricultural University. Nevertheless, the sensory evaluation was conducted in strict accordance with ethical guidelines (*e.g.*, Declaration of Helsinki) to ensure the protection of participants' rights and privacy. All participants were fully informed about the study's purpose, procedures, and any potential risks before participation. Written and verbal informed consent was obtained from each participant before the analysis. Participation was entirely voluntary, and individuals were free to withdraw from the study at any point without penalty. All personal data were treated with strict confidentiality. According to the tea sensory evaluation guidelines (GB/T 23776 2018), the assessment was conducted by a panel of four experienced tea tasters. The evaluation criteria included appearance (25 %), liquor color (10 %), aroma (25 %), taste (30 %), and infused leaf (10 %), utilizing a confidential scoring method. The overall score was calculated as follows: total score = appearance score * 0.25 + liquor color score * 0.1 + aroma score * 0.25 + taste score * 0.3 + infused leaf score * 0.1. Each sample was evaluated and commented on three times. The final score was calculated as the average of the scores provided by the four skilled experts.

#### 2.3.2 Electronic nose analysis

Following the method described by Song et al. (2023), 0.3 g of the sample was accurately weighed and placed into a 20 mL headspace vial. Then, 10 mL of boiling water was added, and the mixture was allowed to soak for 60 min to eliminate water vapor in the vial and ensure gas saturation before measurement. The sensor cleaning time was 110 s, with a zeroing time of 5 s, a sampling interval of 2 s, and a measurement duration of 70 s. Pre-sampling lasts 4 s, and the injection flow rate was maintained at 300 mL/min. The electronic nose (PEN3, AIRSENSE, Germany) features an array of 10 metal oxide sensors, with their performance detailed in Table S1.

#### 2.3.3 Extraction of volatile compounds

To extract volatile substances from tea samples, a modified version of the method was employed (Wang et al., 2023a; Zhai et al., 2022; Zeng et al., 2022). The procedure involved the following steps: freeze-dried tea samples were ground using a pre-frozen mortar and pestle. Then, 0.1 g of the tea powder and 0.2 g of solid sodium chloride were weighed into a 20 mL headspace vial. Then, 5 mL of boiling ultrapure water, a magnetic stir bar, and 10 μL of a 10 mg/L ethyl decanoate internal standard were added. After equilibrating at 60 °C for 15 min under sealed conditions, an aged 50/30 μm CAR/PDMS/DVB extraction fiber was used to adsorb the aroma for 30 min in the headspace vial until equilibrium was reached. Finally, the extraction fiber was inserted into the gas chromatograph injection port and desorbed at 250 °C for 5 min utilizing a splitless injection method.

#### 2.3.4 Analysis of volatile compounds by GC–MS and GC-olfactometry

The GC–MS analysis conditions, adapted from Yao et al. (2023), were as follows: a low-bleed gas chromatography column (DB-5MS, 30 m × 0.25 mm × 0.25 μm) was used for separation, with the injection port temperature set at 250 °C. The initial column temperature was set at 40 °C and held for 5 min, followed by a ramp of 6 °C/min to 130 °C, which was held for an additional 5 min. The temperature then increases at a rate of 6 °C/min to reach 250 °C. Helium gas (purity ≥99.999 %) was the carrier gas at a flow rate of 1 mL/min. The mass spectrometry conditions include electron impact ionization mode with an energy setting of 70 eV, an ion source temperature of 230 °C, a quadrupole temperature of 150 °C, and a mass spectrometry interface temperature of 250 °C. The scan range was set from 35 to 450 amu. The extraction was split into a 1:1 ratio for detection by both the mass spectrometer and the olfactory detector following gas chromatography separation. The airflow rate was set at 60 mL/min (humidified). The olfactory detector (7890-5977B Sniffer, Agilent) is maintained at 180 °C.

#### 2.3.5 Qualitative and quantitative methods

Based on the target peak mass spectral information obtained from GC–MS, a comparison was made with the National Institute of Standards and Technology (NIST) standard spectral library for identification. Retention indices were calculated, and qualitative analysis was performed by comparing these indices with those reported in the literature and relevant databases. Ethyl decanoate was used as an internal standard for relative quantification analysis.

#### 2.3.6 Aroma extract dilution analysis (AEDA) and relative odor activity values (ROAV) calculation

Aroma extract dilution analysis (AEDA) was conducted to assess the intensity of different aroma compounds, and the flavor dilution factor (FD) was evaluated (Wang et al., 2024). The aroma compounds adsorbed by HS-SPME were used to dilute the samples by adjusting the GC inlet split ratio (1:1, 3:1, 7:1, 15:1, *etc.*), three panelists sniffed from low to high dilutions until no odor could be detected. The panelist records the time the odor is sniffed for subsequent identification of the aroma compounds. The final FD is the highest dilution factor at which two or more panelists can sniff the odor.

Additionally, the relative odor activity values (ROAVs) were calculated according to Eq. (1) (Ma et al., 2024; Wang et al., 2024).

ROAV_i_ = C_i_/OT_i_ (Eq. 1).

whereby C_i_ represents the mass concentration of the volatile compound (μg/L), and OT_i_ represents the odor threshold of the volatile compound in water (μg/L).

### 2.4 Data processing and analysis

A single-factor ANOVA test was conducted using IBM SPSS Statistics 26 software, with significance analysis performed through Duncan's multiple comparisons (*P* < 0.05). Principal Component Analysis (PCA) and Orthogonal Projections to Latent Structures Discriminant Analysis (OPLS-DA) were performed on aroma volatile compound data using SIMCA 14.1. PCA analysis for electronic nose data was performed using MetaboAnalyst (www.metaboanalyst.ca). Origin 2021 software and TBtools were also utilized to generate plots and heat maps.

## 3 Results and discussion

### 3.1 Sensory evaluation

**Fig. 2:**
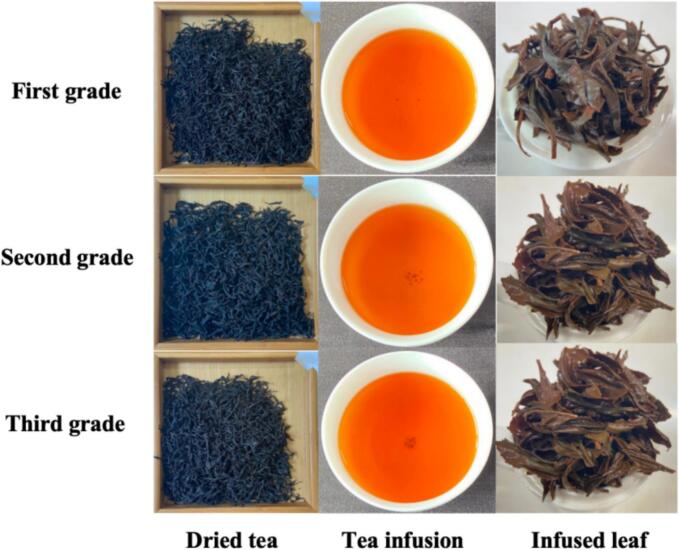
The appearance and infusion of Tanyang Congou black tea with flowery-fruity flavor.

The appearance, liquor color, and infused leaf of Tanyang Congou black tea with flowery-fruity flavor across different grades were shown in Fig. 2. Its appearance features tightly twisted, plump tea leaves that exhibit a dark, lustrous color; the aroma showcases floral, fruity, and honey-like notes; the tea infusion is a bright, clear, and vibrant red, offering a fresh, brisk, and mellow taste that lingers on the palate; the infusion reveals a distinct aroma, while the brewed leaves maintain a bright red hue after steeping. As shown in Table S2, the overall score, as well as appearance, aroma, liquor color, taste, and leaf bottom, diminished as the quality of black tea declined. The third-grade tea received the highest score (90.47), indicating that its flavor quality surpasses that of the second-grade tea (85.87) and the third-grade tea (75.30). The sensory score of black tea is closely linked to the tenderness of fresh leaves (Li et al., 2023). Sensory evaluation indicated that the black tea exhibited a typical aromatic quality characterized by floral and fruity flavors, and complied with the requirements in the standards (DB35/T 632., 2022).

### 3.2 Odor compounds analysis using electronic nose

**Fig. 3:**
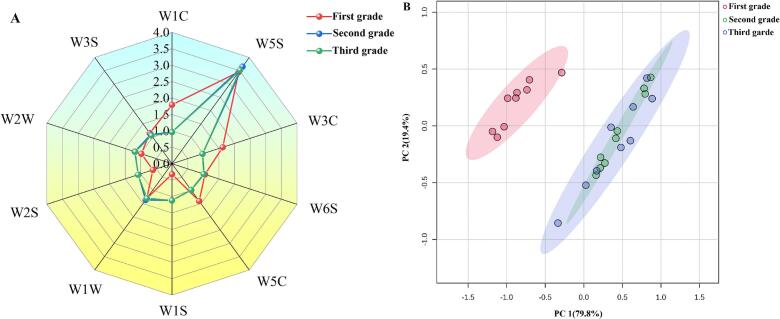
Analysis of Tanyang Congou black tea odor components with flowery-fruity flavor. (A) Electronic nose radar chart; (B) PCA score plot of the electronic nose.

The odor components of Tanyang Congou black tea, characterized by a flowery-fruity flavor, were analyzed using an electronic nose; the radar chart of the electronic nose sensor responses is presented in Fig. 3A. The signal response of sensor W5S (nitrogen oxide) to Tanyang Congou black tea with flowery-fruity flavor was significantly higher than that of other sensors, indicating its sensitivity to nitrogen compounds. Following W5S, the response values were ranked as W1W (sulfide), W1C (aromatic substances, benzene derivatives), W3C (aromatic substances, amines), W5C (short-chain aliphatic aromatic substances), and W2S (alcohols, aldehydes and ketones). All 10 sensors demonstrated response values across samples of different grades. The odor components of the second grade tea and third grade tea were relatively consistent; however, the sensors were unable to differentiate between the second and third grades (Fig. 3B).,The PCA analysis results were aligned with the radar chart of the electronic nose sensor response signals, similar results were obtainted from the black tea discrimination (Yan et al., 2022). The main sensors that highlight the differences in odor composition among the three grades were W5S, W1C, W3, W5C, and W2S.

### 3.3 Aroma profiles of Tanyang congou black tea with flowery-fruity flavor

#### 3.3.1 Analysis of volatile compounds

Three different grades of Tanyang Congou black tea, characterized by a flowery-fruity flavor, were found to contain 70 volatile compounds using HS-SPME coupled with GC–MS. The detailed relative contents of the identified volatiles are listed in Table 1, comprising 8 alcohols, 14 aldehydes, 15 esters, 7 ketones, 21 terpenes, and 5 heterocyclic compounds. Among them, alcohols (695.22 μg/L), aldehydes (610.13 μg/L), and esters (601.67 μg/L) are the main volatile components, accounting for 30.17 %, 26.48 %, and 26.11 % of the total content, respectively. The research results indicated that alcohols and ketones, aldehydes, and esters primarily contributed to the floral aroma, citrus-like and green flavor, and sweet and coconut-like odor of tea (Wang et al., 2024; Zhai et al., 2022). It can be observed that the relative contents of the top volatile compounds in three different grades are geraniol, 2-phenylethanol, (*E*)-nerolidol, 2-hexenal, benzaldehyde, phenylacetaldehyde, citral, methyl salicylate, hexanoic acid geraniol ester, (E)-*β*-ionone, and *β*-laurene (Table 1). It has been reported that geraniol, methyl salicylate, and (*E*)-*β*-ionone are the most important aroma compounds in Keemun, Assam, Darjeeling, and Ceylon black teas (Kang et al., 2019).

**Table 1:** Relative content of volatile compounds in Tanyang Congou black tea with flowery-fruity flavor.

NO	Compounds	CAS	Odor descriptions*	Qualitative method	DB-5MS	RI-nist	Relative content (μg/L)		
							FG	SG	TG
Alcohols									
1	linalool	78–70-6	Citrus-like, flowery	MS, RI, O	1100	1101	8.21 ± 0.97^a^	7.26 ± 3.83^a^	7.74 ± 0.87^a^
2	3,5-octadien-2-ol	69,668–82-2	/	MS, RI	1073	1090	0.97 ± 0.17^a^	1.46 ± 0.82^a^	ND
3	2-phenylethanol	60–12-8	Flowery, honey-like	MS, RI, O	1121	1112	36.73 ± 6.14^a^	35.62 ± 13.23^a^	29.23 ± 8.38^a^
4	(*E*)-linalool oxides (pyranoid)	39,028–58-5	Woody	MS, RI, O	1176	1173	1.25 ± 0.13^a^	ND	ND
5	geraniol	106–24-1	Rose-like, citrus-like	MS, RI, O	1247	1256	179.22 ± 5.59^a^	147.28 ± 28.80^ab^	120.12 ± 29.44^b^
6	(E)-nerolidol	40,716–66-3	Woody, flowery	MS, RI, O	1563	1562	16.98 ± 2.32^b^	41.62 ± 12.45^a^	36.18 ± 8.96^ab^
7	cypress alcohol	77–53-2	Sweet	MS, RI, O	1615	1607	1.27 ± 0.23^a^	0.85 ± 0.25^ab^	0.58 ± 0.10^b^
8	benzyl alcohol	100–51-6	Bitter almond-like, fruity	MS, RI	1043	1030	ND	1.65 ± 1.30^a^	ND
Aldehydes									
9	hexanal	66–25-1	Green, grassy	MS, RI, O	802	801	7.68 ± 1.10^a^	13.08 ± 1.53^b^	7.99 ± 0.38^a^
10	(E)-2-hexenal	6728-26-3	Green apple-like, bitter almond-like	MS, RI, O	855	850	12.03 ± 3.49^b^	21.16 ± 4.83^a^	14.37 ± 1.87^ab^
11	heptanal	111–71-7	Citrus-like, fatty	MS, RI, O	904	901	1.22 ± 0.10^a^	2.17 ± 0.40^a^	1.62 ± 0.79^a^
12	2-heptenal	57,266–86-1	Green, fatty	MS, RI, O	961	958	1.27 ± 0.41^b^	2.97 ± 1.14^a^	1.54 ± 0.62^ab^
13	benzaldehyde	100–52-7	Bitter almond-like, marzipan-like	MS, RI, O	966	964	44.49 ± 3.64^a^	57.80 ± 18.17^a^	53.78 ± 4.71^a^
14	(E,E)-2,4-heptadienal	4313-03-5	Fatty, flowery	MS, RI, O	1022	995	6.31 ± 2.66^b^	25.23 ± 9.48^a^	6.40 ± 1.91^b^
15	phenylacetaldehyde	122–78-1	Honey-like, beeswax-like	MS, RI, O	1047	1051	64.69 ± 8.13^a^	62.01 ± 19.58^a^	74.46 ± 12.27^a^
16	nonanal	124–19-6	Citrus-like, soapy	MS,RI,O	1105	1102	3.74 ± 0.37^b^	15.35 ± 2.17^a^	4.17 ± 2.98^b^
17	*β*-cyclcitral	432–25-7	Tropical, saffron, herbal, tobacco-like	MS,RI,O	1219	1217	1.69 ± 0.05^a^	3.01 ± 1.31^a^	1.79 ± 0.60^a^
18	(Z)-3,7-dimethyl-2,6-octadienal	106–26-3	Citrus-like, fruity	MS,RI,O	1237	1238	2.87 ± 0.47^a^	3.47 ± 0.78^a^	2.99 ± 1.16^a^
19	citral	5392-40-5	Fruity	MS,RI,O	1265	1240.5	27.85 ± 1.70^a^	18.17 ± 4.13^b^	15.19 ± 2.74^b^
20	(E)-2-butyl-2-octenal	13,019–16-4	/	MS,RI	1370	1378	0.97 ± 0.33^a^	ND	ND
21	(E)-2-octenal	2548-87-0	Fatty, nutty	MS,RI	1060	1062	ND	5.15 ± 0.91^b^	3.04 ± 0.35^a^
22	2,4-dimethylbenzaldehyde	15,764–16-6	/	MS,RI	1181	1181	ND	3.2 ± 1.55^a^	1.47 ± 0.84^a^
Esters									
23	(E)-hex-3-enyl butanoate	53,398–84-8	/	MS,RI	1184	1185	7.87 ± 0.17^a^	8.66 ± 1.36^a^	10.49 ± 2.56^a^
24	methyl salicylate	119–36-8	Mint-like	MS,RI,O	1195	1193	163.34 ± 9.35^a^	122.91 ± 19.59^b^	108.90 ± 3.46^b^
25	(Z)-3-hexen-2-yl butanoate	53,398–85-9	Herb, sweet	MS,RI,O	1227	1233.5	3.38 ± 0.64^b^	5.34 ± 0.95^a^	4.61 ± 0.57^ab^
26	geranyl acetate	1189-09-9	/	MS,RI	1319	1321	1.73 ± 0.03^a^	ND	ND
27	hex-3-enyl hexanoate	84,434–19-5	/	MS,RI	1374	1376	0.59 ± 0.03^a^	ND	ND
28	hexanoic acid geraniol ester	31,501–11-8	Fruity	MS,RI,O	1378	1376	26.12 ± 3.46^b^	47.86 ± 9.73^a^	36.57 ± 2.91^ab^
29	hexyl hexanoate	6378-65-0	Fruity	MS,RI,O	1383	1384	5.10 ± 0.47^a^	5.99 ± 1.87^a^	5.49 ± 0.87^a^
30	N-hexanoic acid ((E)-2-hexenyl) ester	53,398–86-0	/	MS,RI	1387	1391	3.59 ± 0.72^a^	4.01 ± 1.67^a^	3.35 ± 1.76^a^
31	isobutyric acid phenethyl ester	103–48-0	/	MS,RI	1447	1449	3.90 ± 0.17^a^	ND	ND
32	2-methylbutyric acid phenethyl ester	24,817–51-4	/	MS,RI	1488	1488	6.51 ± 0.72^c^	54.52 ± 11.46^a^	26.15 ± 3.74^b^
33	(Z)-3-hexen-1-ol benzoate	25,152–85-6	/	MS,RI	1583	1567	1.20 ± 0.04^a^	ND	4.21 ± 1.71^b^
34	2,2,4-trimethyl-1,3-pentanediol diisobutyrate	6846-50-0	/	MS,RI	1587	1587.5	1.44 ± 0.12^a^	ND	1.17 ± 0.14^a^
35	butyric acid phenethyl ester	103–52-6	/	MS,RI	1220	1219	ND	24.79 ± 5.02^a^	ND
36	dihydroactinidiolide	17,092–92-1	Musty, pungent	MS,RI	1533	1522	ND	1.86 ± 1.21^a^	ND
37	methyl jasmonate	042536–97-0	Jasmine-like, flowery	MS,RI	1654	1684.5	ND	3.44 ± 1.65^a^	ND
Ketones									
38	jasmone	488–10-8	Flowery	MS,RI	1397	1394	1.26 ± 0.26^c^	10.38 ± 0.83^a^	5.87 ± 0.88^b^
39	*α*-ionone	127–41-3	Flowery, violet-like	MS,RI,O	1424	1424	1.08 ± 0.35^b^	1.39 ± 0.47^ab^	2.31 ± 0.52^a^
40	geranyl acetone	3796-70-1	Flowery	MS,RI	1452	1428	3.13 ± 0.46^a^	2.80 ± 0.09^a^	2.31 ± 0.52^a^
41	(E)-*β*-ionone	79–77-6	Flowery, violet-like	MS,RI,O	1481	1485	15.29 ± 3.30^ab^	18.41 ± 3.79^a^	11.51 ± 0.21^b^
42	6-methyl-5-hepten-2-one	110–93-0	Citrus-like, fruity	MS,RI,O	984	986	ND	6.88 ± 1.07 ^a^	4.42 ± 1.10 ^a^
43	3,5-octadien-2-one	38,284–27-4	/	MS,RI	1075	1090	ND	5.36 ± 3.11^a^	ND
44	2-methyl-6-methylene-1,7-octadien-3-one	41,702–60-7	/	MS,RI	1319	1345	ND	3.07 ± 1.26^a^	ND
Olefins									
45	*β*-laurene	123–35-3	Fatty	MS,RI,O	989	988	18.35 ± 1.34^a^	11.91 ± 3.44^b^	10.43 ± 0.87^b^
46	pinene	586–63-0	Pine woody	MS,RI,O	1017	1055	0.75 ± 0.12^a^	ND	ND
47	(+)-dipentene	5989–27-5	Citrus-like, carrot-like	MS,RI	1030	1030	2.94 ± 0.30^a^	1.75 ± 0.91^a^	1.91 ± 0.15^a^
48	(*E*)-*Β*-basilene	3779-61-1	Terpene-like	MS,RI,O	1036	1044	3.48 ± 0.54^a^	2.20 ± 1.23^a^	2.54 ± 0.98^a^
49	2,4-dimethyl styrene	1195-32-0	/	MS,RI	1092	1089	1.47 ± 0.28^a^	1.24 ± 0.43^a^	0.94 ± 0.36^a^
50	isoborneol	7216-56-0	/	MS,RI,O	1129	1131	2.55 ± 0.81^a^	ND	ND
51	tetradecane	629–59-4	/	MS,RI	1398	1399	0.75 ± 0.12^a^	ND	ND
52	(*E*)-*β*-acacia olefin	18,794–84-8	/	MS,RI	1454	1460	2.47 ± 0.13^b^	7.58 ± 2.87^a^	4.62 ± 1.18^ab^
53	pentadecane	629–62-9	/	MS,RI	1499	1497	0.66 ± 0.11^a^	ND	ND
54	*α*-farnesene	502–61-4	Citrus-like, flowery, Woody	MS,RI,O	1504	1504	1.93 ± 0.93^c^	8.41 ± 0.57^a^	4.76 ± 0.99^b^
55	*β*-copaene	495–61-4	/	MS,RI	1508	1510	0.60 ± 0.17^a^	1.61 ± 0.79^a^	0.94 ± 0.48^a^
56	Δ-juniperene	483–76-1	/	MS,RI	1520	1523	0.25 ± 0.07^b^	0.78 ± 0.34^a^	0.79 ± 0.05^a^
57	Α-dihydrocalamenene	21,391–99-1	/	MS,RI	1545	1541	0.25 ± 0.06^a^	ND	ND
58	hexadecane	544–76-3	/	MS,RI	1599	1599	1.12 ± 0.23^a^	ND	0.86 ± 0.28^a^
59	newly synthesized diene	504–96-1	/	MS,RI	1838	1836	0.52 ± 0.12^a^	ND	ND
60	5-ethyl-m-xylene	934–74-7	/	MS,RI	1025	1059	ND	3.01 ± 1.28^a^	2.47 ± 0.44^a^
61	Β-nitrophenylethane	6125-24-2	/	MS,RI	1303	1300	ND	41.77 ± 8.72^a^	ND
62	α-cedrene	469–61-4	/	MS,RI	1503	1504	ND	0.61 ± 0.34^a^	ND
63	*α*-caryophyllene	25,532–79-0	/	MS,RI	1542	1544.6	ND	0.72 ± 0.26^a^	ND
64	styrene	100–42-5	/	MS,RI	890	886	1.01 ± 0.15^a^	ND	ND
65	(E)-calamenene	73,209–42-4	/	MS,RI	1524	1530	0.57 ± 0.11^a^	ND	ND
Heterocycles									
66	*(E)*-2-(2-pentenyl)furan	3208-16-0	/	MS,RI	999	1001	1.30 ± 0.44^a^	ND	ND
67	phenylacetonitrile	140–29-4	/	MS,RI	1145	1150	26.79 ± 3.52^c^	126.90 ± 16.60^a^	60.21 ± 3.14^b^
68	naphthalene	91–20-3	/	MS,RI	1191	1186	5.86 ± 1.97^a^	3.42 ± 0.68^a^	4.46 ± 0.92^a^
69	indole	120–72-9	Fecal-like, mothball-like	MS,RI	1343	1327	ND	21.15 ± 2.23^a^	ND
70	(Z)-2-(2-pentenyl)furan	70,424–14-5	/	MS,RI	999	1001	ND	0.54 ± 0.29^a^	ND

Note: RI represents the retention index of volatile compounds, calculated based on the retention times of volatile compounds on a DB-5MS capillary column and the retention times of n-alkanes ranging from C7 to C40; RI-nistrefers to the retention index as represented in the NIST mass spectrometry database; MS、RI and O represent qualitative methods of NIST Mass Spectrometry Database, Retention Index and Olfactory, respectively; *: Odor descriptions are obtained from the literature, flavornet database or odorant database, /: no odor descriptions were found; ND: not detected; FG (first grade), SG (second grade), TG (third grade). The different letters indicate significant differences at *P* < 0.05.

#### 3.3.2 PCA and OPLS-DA analysis

**Fig. 4:**
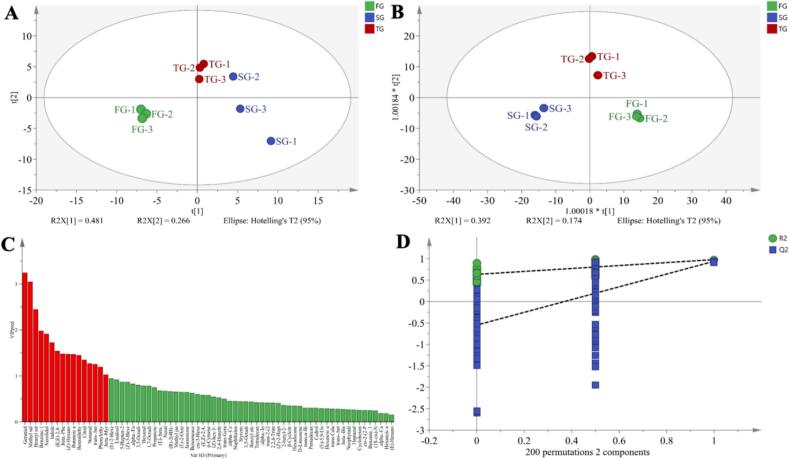
PCA and OPLS-DA analysis of Tanyang Congou black tea with flowery-fruity flavor. (A) PCA score graph; (B) OPLS-DA score plot; (C) Variable importance values of OPLS-DA; (D) 200 permutation tests.

Unsupervised Principal Component Analysis (PCA) was performed on the GC–MS-identified volatile compounds. As shown in Fig. 4A, the three Tanyang Congou black tea grades with flowery-fruity flavor were effectively distinguished with R^2^X = 0.846 and Q^2^ = 0.406 model parameters. Subsequently, supervised orthogonal partial least squares discriminant analysis (OPLS-DA) was conducted based on the PCA results, with model parameters of R^2^X = 0.948, R^2^Y = 0.974, and Q^2^ = 0.887. Fig. 4B showed that the three grades of Tanyang Congou black tea with their flowery-fruity flavor were more distinctly separated, indicating significant differences in the volatile compounds among the various tea grades.

Variable Importance in the Projection (VIP) in the OPLS-DA model characterizes the differential contributions of compounds in the samples. A VIP value greater than 1 indicates that a compound makes a significant contribution to the differences between the samples (Wei et al., 2023). Through OPLS-DA analysis, 16 differential volatile compounds with VIP values greater than 1 have been analyzed (Fig. 4C). These compounds included linalool, methyl salicylate, benzonitrile, (*E*)-linalool oxide, indole, phenethyl benzoate, *β*-nitrostyrene, (E,E)-2,4-heptadienal, hexyl salicylate, 2-methylbut-2-yl benzoate, benzaldehyde, citral, nonanal, (E)-*β*-ionone, 2-phenylethanol, and *β*-caryophyllene. Additionally, 200 permutation tests were conducted to assess the reliability of the OPLS-DA model. The intercept for R^2^ is 0.633, and the intercept for Q^2^ is −0.55, indicating no overfitting for the model. This demonstrated that the model exhibits high credibility and predictability (Fig. 4D). It is well known that each aroma compound in tea presents a unique aroma profile with varying sensory thresholds. Merely having a high relative content of aromatic compounds does not guarantee a significant contribution to the overall aroma quality of the tea. Therefore, to identify the key aroma compounds in Tanyang Congou black tea with a flowery-fruity flavor, it is necessary to conduct relative odor activity value calculations and aroma extract dilution analysis, coupled with GC-O-MS analysis.

### 3.4 Key aroma compounds of Tanyang congou black tea with flowery-fruity flavor

#### 3.4.1 AEDA and GC-O-MS analysis

**Fig. 5:**
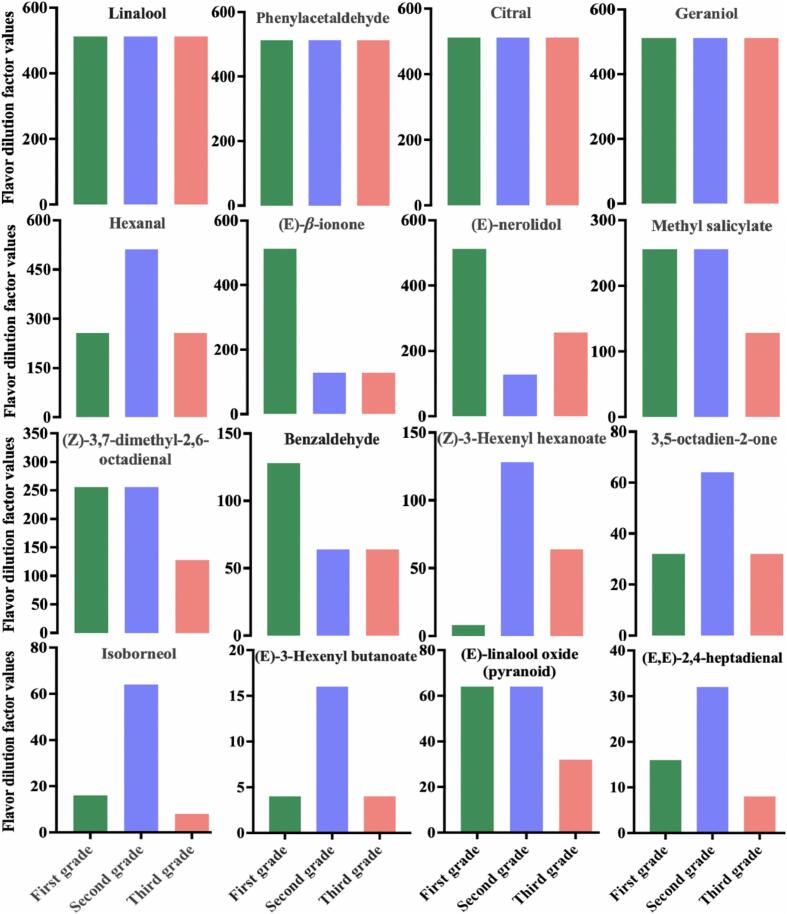
16 volatiles (FD ≥ 8) in Tanyang Congou black tea with flowery-fruity flavor by GC–O–MS and AEDA.

The AEDA and GC-O-MS analysis was carried out to further identify key aroma compounds of Tanyang Congou black tea with flowery-fruity flavor. A total of 29 aroma compounds were identified across all three grades, among them, 16 aroma volatiles, such as geraniol, (*E*)-linalool oxide (pyranoid), linalool, (E)-nerolidol, hexanal, benzaldehyde, (E,E)-2,4-heptadienal, phenylacetaldehyde, (E)-3-hexenyl butanoate, (Z)-3-hexenyl hexanoate, 3,5-octadien-2-one, (Z)-3,7-dimethyl-2,6-octadienal, citral, (E)-*β*-ionone, methyl salicylate, and isoborneol have higher FD values over 8 in all three black tea grades (Fig. 5) and were selected as key aroma compounds. The higher the FD values of compounds, the greater their contribution to the tea aroma (Wang et al., 2024). Linalool (512, 512, 512; citrus-like, flowery), phenylacetaldehyde (512, 512, 512; honey-like, beeswax-like), citral (512, 512, 512; fruity) and geraniol (512, 512, 512; rose-like, citrus-like) were the most intensely perceived in Tanyang Congou black tea with flowery-fruity flavor, followed by hexanal (256, 512, 256; green, grassy), (*E*)-*β*-ionone (512, 128, 128; flowery, violet-like) and (E)-nerolidol (512, 128, 128; woody, flowery), methyl salicylate (256, 256, 128; mint-like) and (Z)-3,7-dimethyl-2,6-octadienal (256, 256, 128; citrus-like, fruity), benzaldehyde (128, 64, 64; bitter almond-like, marzipan-like) and (Z)-3-Hexenyl hexanoate (8, 128, 64, fruity), and the other 5 aroma compounds also received strongly positive response (FD ≥ 8). These aroma compounds are likely essential components that contribute to the overall aroma profile of Tanyang Congou black tea, characterized by a flowery-fruity flavor. The FD values of (*E*)-*β*-ionone, (E)-nerolidol, benzaldehyde, and (E)-linalool oxide (pyranoid) in first-grade black tea were higher than those in second- and third-grade teas. This could be a primary reason for the superior floral and fruity aroma of first-grade tea compared to second and third-grade teas, and the results were consistent with the sensory evaluation.

#### 3.4.2 Screening for key aroma compounds and ROAV calculation

A high concentration of volatiles does not necessarily indicate a significant contribution. Herein, the volatile compounds identified by GC–MS were further quantified using ROAV calculation, the larger the ROAV, the greater the contribution to the tea aroma. As shown in Table 2, 11 compounds with ROAV >1 were calculated, including geraniol, linalool, (*E*)-nerolidol, hexanal, phenylacetaldehyde, nonanal, citral, (E,E)-2,4-heptadienal, (E)-*β*-ionone, *α*-ionone and *β*-laurene.

**Table 2:** Aroma compounds (relative odor activity value ≥1) and odor attributes of Tanyang Congou black tea samples.

NO	Compounds	Odor descriptions*	Threshold values (μg/L)	ROAV		
				FG	SG	TG
1	Geraniol	Rose-like, citrus-like	3.2	56.00 ± 1.74^a^	46.02 ± 9.00^ab^	37.53 ± 9.20^b^
2	Linalool	Citrus-like, flowery	0.6	13.68 ± 1.62^b^	12.11 ± 6.38^b^	23.52 ± 8.43^a^
3	(E)-nerolidol	Woody, flowery	10	1.69 ± 0.23^b^	5.67 ± 2.03^a^	3.61 ± 0.89^ab^
4	Hexanal	Green, grassy	2.4	3.20 ± 0.45^a^	6.45 ± 2.00^a^	4.74 ± 2.45^a^
5	Phenylacetaldehyde	Honey-like, beeswax-like	5.2	12.44 ± 1.93^a^	17.57 ± 9.79^a^	14.31 ± 2.36^a^
6	Nonanal	Citrus-like, soapy	2.8	1.33 ± 0.13^b^	5.48 ± 0.77^a^	1.49 ± 1.06^b^
7	Citral	Fruity	5	27.85 ± 1.70^a^	3.63 ± 0.82^b^	3.03 ± 0.54^b^
8	(E,E)-2,4-heptadienal	Fatty, flowery	0.032	197.49 ± 83.37^b^	788.47 ± 96.32^a^	280.85 ± 56.3^b^
9	(E)-*β*-ionone	Flowery, violet-like	0.021	728.43 ± 57.21^a^	625.05 ± 54.74^a^	407.79 ± 43.28^a^
10	*α*-ionone	Flowery, violet-like	0.4	2.70 ± 0.88^a^	3.47 ± 1.19^a^	2.83 ± 1.05^a^
11	*β*-laurene	Fatty	1.2	15.29 ± 1.12^a^	9.92 ± 2.86^b^	8.69 ± 0.72^b^

Note: FG (first grade), SG (second grade), TG (third grade). The different letters indicate significant differences at *P* < 0.05. *: Odor descriptions are obtained from the literature, flavornet database or odorant database.

**Fig. 6:**
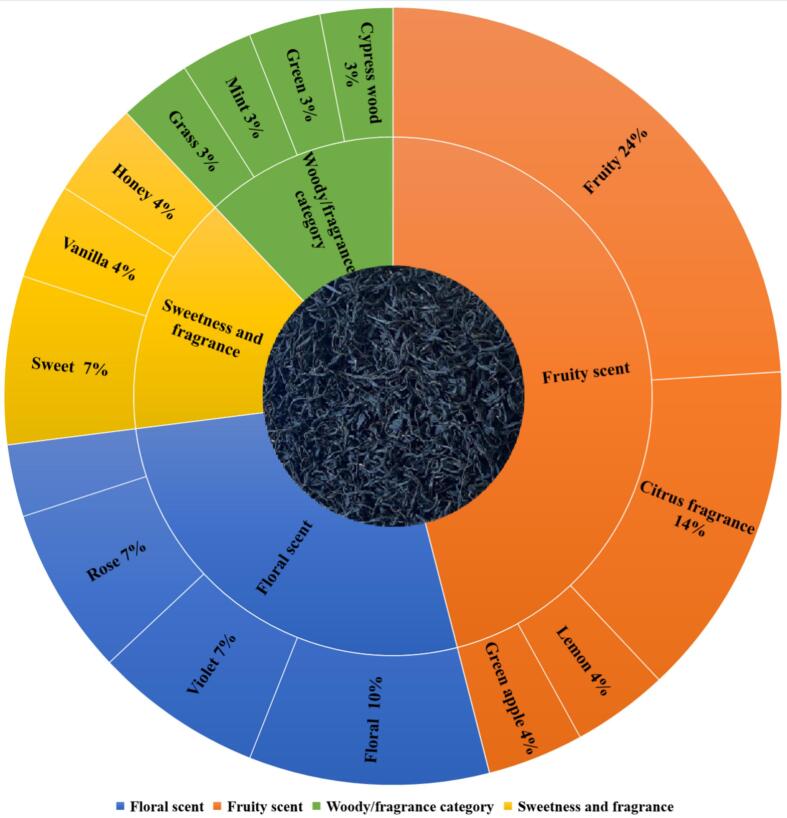
The aroma wheel of Tanyang Congou black tea with flowery-fruity flavor.

(E)-*β*-ionone (flowery, violet-like) presented the highest ROAV values (> 400), followed by (E,E)-2,4-heptadienal (fatty, flowery, > 100), linalool (citrus-like, flowery), geraniol (rose-like, citrus-like) and phenylacetaldehyde (honey-like, beeswax-like,10–100), and the OAVs of other 6 compounds, *i.e.*, (E)-nerolidol (woody, flowery), hexanal (green, grassy), nonanal (citrus-like, soapy), citral (fruity), *α*-ionone (flowery, violet-like) and *β*-laurene (fruity) were 1–10. The ROAVs of (*E*)-*β*-ionone, citral and geraniol in the first grade were higher than that of the second- and third-grade teas (Table 2), which was consistent with the results obtained from the sensory evaluation and AEDA analysis. Overall, (*E*)-*β*-ionone, (E)-nerolidol, geraniol, citral, linalool, hexanal, and phenylacetaldehyde are characteristic aromatic compounds contributing to the “floral and fruity” aroma profile of Tanyang Congou black tea based on ROAV and FD. Aromatic profiles were categorized for the aroma compounds identified by GC-O analysis, and the aromatic flavor wheel for Tanyang Congou black tea with flowery-fruity flavor was shown in Fig. 6. Floral and fruity are predominantly characterized, and aroma compounds constitute a significant proportion, accounting for 27 % and 46 %, respectively. Fruity flavor characteristics of black tea prepared from *Camellia sinensis* (L.) O. Kuntze *cv.* Jinmudan were identified in previous study, and (*Z*)-methyl epi-jasmonate, phenylacetaldehyde, linalool, geraniol and jasmine lactone were the main aroma active compounds (Li et al., 2022). For the Congou black tea using traditional manufacturing methods, floral flavor did not manifest and linalool, (Z)-pyran linalool oxide, (*E*)-furan linalool oxide, *β*-laurene, methyl salicylate, and 2-phenylethanol were identified as key contributors to the aromatic flavors (Mao et al., 2018). Shaking is an innovative technology employed in black tea manufacturing to enhance floral flavor (Wang et al., 2024), incorporating the shaking in the early withering process into Hunan black tea can promote the intensities of indole and methyl jasmonate, contributing to the formation of floral aromas (Yin et al., 2023). Shaking and standing treatment significantly improved the floral and sweet quality of summer black tea, with high ROAVs of linalool, (*E*)-*β*-ionone, geraniol, *β*-myrcene, (E)-2-hexenal, phenylacetaldehyde, (Z)-3-hexenyl hexanoate, 1-hexanol, and 2-phenylethanol (Wang et al., 2024). Eight key differential aromatic odorants, *i.e.*, geraniol, citral, (*E*)-nerolidol, 2,6-dimethyl-2,4,6-octantriene, (Z)-hexanoic acid-3-hexenyl ester, ethyl hexanoate, decanal, and (E)-*β*-ionone, were identified in shaking treatment black tea (Wang et al., 2022a).

### 3.5 The possible mechanism of floral and fruity aroma during manufacturing

An essential factor in the formation of tea aroma lies in aroma precursors, which undergo complex chemical reactions and biosynthetic pathways during tea manufacturing, such as carotenoid oxidation and degradation, fatty acid oxidation and degradation, glycoside hydrolysis, Maillard reaction, and biosynthesis of volatile terpenoids (Chen et al., 2022; Zhai et al., 2022). Shaking technology enhances cell fragmentation by flipping the leaves, which increases the contact between enzymes like oxidase and hydrolase and their substrates, as well as biochemical reaction induced by abiotic stress. This process promotes the oxidation of polyphenols, the hydrolysis of glycosides and secondary metabolic biosynthesis, contributing to the formation of floral and fruit aromas (Wang et al., 2023a; Wang et al., 2024; Hu et al., 2018). The manufacturing samples of Tanyang Congou black tea with flowery-fruity flavor at different stages, including fresh leaf (FL), withering (W), shaking (S), rolling (R), fermention (F), drying (D), and re-drying (ReD), were collected, respectively. HS-SPME-GC–MS analysis was used to investigate the key aromatic compounds to elucidate the possible mechanism of formation of floral and fruity aroma compounds. Floral and fruity aroma profiles are generally associated with four kinds of pathways, *i.e.*, terpenoid biosynthesis pathway ((*E*)-geraniol, linalool, citral, geraniol), fatty acid derivative pathway (hexanal), carotenoid derivative pathway ((E)-*β*-ionone), and volatile phenylpropane/benzenoid pathway (phenylacetaldehyde) (Chen et al., 2019; Chen et al., 2022; Yang et al., 2013), as shown in Fig. S1 – Supplementary materials.

**Fig. 7:**
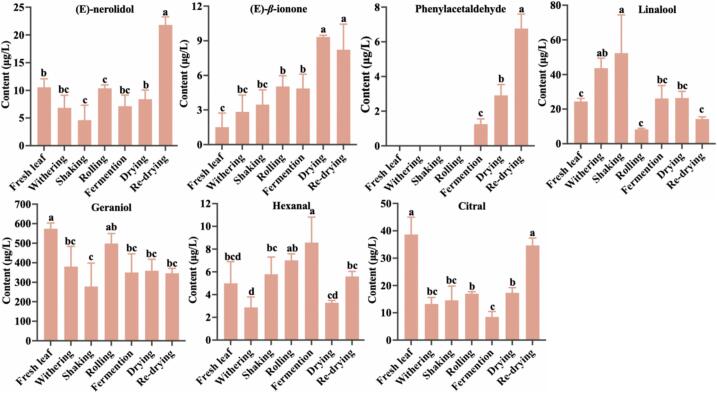
The content changes of key aromatic compounds during the manufacturing.

As illustrated in Fig. 7, the contents of (*E*)-nerolidol, (E)-*β*-ionone, and phenylacetaldehyde were significantly increased during the tea manufacturing (*P* < 0.05). *β*-carotene cleavage dioxygenases catalyze the oxidation of *β*-carotene (Chen et al., 2024; Wang et al., 2020), leading to the continuous accumulation of (E)-*β*-ionone during withering and subsequent tea manufacturing stages. Phenylacetaldehyde is a volatile compound that is produced from phenylalanine *via* a deamination reaction. From the fermenting to the re-drying, there is a significant increase in the content of phenylacetaldehyde (*P* < 0.05). This could be attributed to the intense deamination reaction of phenylalanine under heating conditions, causing a rapid conversion of phenylalanine to phenylacetaldehyde (Ho et al., 2015). During the manufacturing, the relative content of linalool initially increased and then declined, peaking during the shaking. At this stage, the cell walls of the tea leaves are nearly intact, and endogenous hydrolytic enzymes and bound glycosides would not undergo hydrolysis due to the different locations. Therefore, linalool is likely not formed by the hydrolysis of its glycosides, but rather synthesized *de novo* (Zeng et al., 2017). Geraniol has the highest relative content and peaks during the rolling through geraniol glycosidase hydrolysis (*P* < 0.05). Tea leaf cell walls are minimally damaged, allowing hydrolytic enzymes and glycosides sufficient contact to interact effectively (Cui et al., 2016; Zeng et al., 2017). Endogenous glycosides are hydrolyzed to release a large amount of geraniol (*P* < 0.05). The content of hexanal significantly increased (*P* < 0.05) after shaking. Mechanical damage induced by the shaking process activated the expression of structural genes in the lipoxygenase pathway of unsaturated fatty acids (Hu et al., 2018). Additionally, dehydration stress on the leaves triggers enhanced fatty acid metabolism during withering, promoting lipoxygenase-mediated lipid oxidation (Wang et al., 2019). Remarkably, the re-drying process significantly increases the content of (*E*)-geraniol, citral, and phenylacetaldehyde, contributing to the development of the floral and fruity aroma profile in Tanyang Congou black tea and enhancing its aromatic quality. Studies have shown that drying step played a crucial role in the formation of sweet and fruity aromas for black tea because of the Maillard reaction (Ma et al., 2024; Qu et al., 2019), aroma compunds of hexanal, 1-hexanol, (Z)-3-hexen-1-ol, (E,E)-2,4-heptadienal, methyl salicylate and *β*-damascenone with ROAVs >10 were found to have increased significantly (*P* < 0.01) after drying (Ma et al., 2024).

Numerous methods have been implemented to explore the feasibility of aroma enhancement of black tea, including withering technology (Huang et al., 2022), light irradiation during withering (Li et al., 2022), fermentation time optimization (Wang et al., 2021), processing technology (Chen et al., 2023; Chen et al., 2024) and external *β*-glucosidase assistance during fermentation (Supriyadi et al., 2021). Sun withering significantly improved the floral and fruity aroma of Keemun black tea with high ROAV values of linalool and geraniol (Huang et al., 2022). Red light significantly increased the activity of glycosidase and expression of *LIS*, *FAS*, *LOX* and *ADH* during withering stage, improving the main floral aroma components of summer-autumn black tea (Li et al., 2022). 3 h was the optimal fermentation time for Yunnan Congou black tea with most abundant of aroma compounds (linalool, phenylacetaldehyde, methyl salicylate, benzaldehyde, 2-pentyl-furan, and (*E*)-*β*-ionone) (Wang et al., 2021). The addition of external *β*-glucosidase during fermentation significantly increased the precursors of linalool, geraniol, and methyl salicylate (Supriyadi et al., 2021). *Re*-rolling processing technology are beneficial to enriching small-leaf Congou black tea aroma, because it significantly increased the levels of aldehydic and ester fatty acid-derived volatiles, amino acid-derived volatiles, carotenoid-derived volatiles, and alkene volatile terpenoid (Chen et al., 2023). In comparison to these methods, shaking technology is straightforward and does not require specific types of tea leaves, but it effectively stimulate endogenous biochemical reaction and generate a higher concentration of aroma compounds or precursors.

## 4 Conclusions

In this study, the aroma profiles of Tanyang Congou Black Tea with a characteristic flowery-fruity flavor were systematically analyzed using sensory evaluation, an electronic nose (*E*-Nose), and headspace solid-phase microextraction gas chromatography-olfactometry-mass spectrometry (HS-SPME-GC-O-MS) . The sensory evaluation confirmed the presence of distinct floral and fruity attributes, validating the tea's preparation process. A total of 29 aroma compounds were identified, among which 16 volatiles exhibited flavor dilution values greater than 8, and 11 compounds had relative odor activity values exceeding 1. Seven key aroma-contributing compounds were identified: (E)-*β*-ionone, (E)-nerolidol, geraniol, citral, linalool, hexanal, and phenylacetaldehyde. These findings provide a comprehensive understanding of the aroma composition of Tanyang Congou Black Tea and offer a theoretical foundation for optimizing the manufacturing of high-aroma black teas.

## CRediT authorship contribution statement

**Di Zhou:** Writing – original draft, Methodology, Investigation, Conceptualization. **Xin-yu Liu:** Writing – original draft, Methodology, Formal analysis. **Miao-qin Xie:** Writing – original draft, Methodology, Investigation, Formal analysis. **Hao-jie Xu:** Writing – original draft, Methodology, Investigation. **Huai-hui Yi:** Writing – original draft, Methodology, Investigation. **Da-xiang Li:** Writing – original draft, Methodology, Investigation. **Ru-yan Hou:** Writing – original draft, Methodology, Formal analysis. **Hui-mei Cai:** Writing – original draft, Project administration, Methodology, Investigation, Conceptualization. **Xiao-chun Wan:** Writing – original draft, Methodology, Investigation, Conceptualization. **Daniel Granato:** Writing – original draft, Conceptualization. **Chuan-yi Peng:** Writing – original draft, Supervision, Project administration, Methodology, Investigation, Conceptualization.

## Declaration of competing interest

We confirm that the work described in this submission has not been published previously. The manuscript has been read and approved by all named authors, as well as by the relevant authorities at the institutions where the research was conducted. We also confirm that all authors have agreed upon the order of authors listed in the manuscript. The authors declare that they have no known competing financial interests or personal relationships that could be perceived as influencing the work reported in this paper. We have carefully considered the protection of intellectual property related to this work and confirm that there are no obstacles to publication, including timing issues concerning intellectual property. We affirm that we have adhered to the regulations of our institution regarding intellectual property. Additionally, no animal studies are involved in this manuscript.

## Data Availability

Data will be made available on request.

## Appendix A Supplementary data

Supplementary material. Image 1
