# Supplementary material for: Characterization of aroma profiles of Tanyang Congou black tea with flowery-fruity flavor: Insights from sensory evaluation and HS-SPME-GC-O-MS
Source: Food Chem X. 2025 May 28;28:102595. doi: 10.1016/j.fochx.2025.102595 (PMC12163174; doi:10.1016/j.fochx.2025.102595)
Supplement: Supplementary file 1 — Supplementary material. [file mmc1.docx]

**Table S1:** Sensitive substances of PEN3 electronic nose sensor.

| NO | Sensors | Features of sensitive compounds |
| --- | --- | --- |
| S1 | W1C | aromatic substances, benzene derivatives |
| S2 | W5S | nitrogen oxide |
| S3 | W3C | aromatic substances, amines |
| S4 | W6S | hydride |
| S5 | W5C | short-chain aliphatic aromatic substances |
| S6 | W1S | alkane |
| S7 | W1W | sulfide |
| S8 | W2S | alcohols, aldehydes and ketones |
| S9 | W2W | aromatic substances, organic sulfide |
| S10 | W3S | long-chain alkanes |

**Table S2:** Sensory evaluation of Tanyang Congou black tea with flowery-fruity flavour.

| Grade | No. | Appearance（25%） | | Aroma（25%） | | Liquor color（10%） | | Taste（30%） | | Leaf bottom（10%） | | Overall score |
| --- | --- | --- | --- | --- | --- | --- | --- | --- | --- | --- | --- | --- |
|  |  | Comment | Score | Comment | Score | Comment | Score | Comment | Score | Comment | Score |  |
| FG | 1 | tight, dark, lustrous, and uniform | 91.0 | sweet aroma with a hint of milk | 90.0 | bright orange-red and clear | 89.0 | sweet and mellow, with floral and fruity | 90.0 | soft and tender, with a uniform red color | 89.0 | 90.1 |
|  | 2 | tight and compact, with a pointed tip, dark, lustrous, and uniform | 93.0 | sweet aroma with prominent floral and fruity | 92.0 | bright orange-red and clear | 89.5 | rich and mellow, with floral and fruity | 91.5 | soft and tender, with a uniform red color and thick leaves | 92.0 | 91.9 |
|  | 3 | tight, dark, lustrous, and uniform | 90.0 | sweet aroma with floral and fruity | 89.0 | bright orange-red | 88.5 | sweet and mellow, with floral and fruity | 89.5 | soft and tender, with a uniform red color | 89.5 | 89.4 |
| SG | 1 | tight and compact, dark and lustrous, relatively uniform, and reasonably clean | 87.0 | floral and fruity are quite prominent | 87.0 | bright orange-red | 86.0 | relatively rich and mellow, with floral and fruity | 88.0 | relatively uniform red color | 87.0 | 87.2 |
|  | 2 | relatively tight and compact, dark and lustrous, reasonably uniform, and relatively clean | 85.0 | floral notes are quite prominent | 84.5 | bright orange-red | 86.0 | smooth and mellow, with floral and fruity | 85.5 | still bright and uniform red color | 85.0 | 85.1 |
|  | 3 | relatively tight and compact, dark and lustrous, and reasonably uniform | 85.5 | has floral and fruity | 84.0 | bright orange-red | 86.5 | smooth and mellow, with floral and fruity | 86.0 | still bright and uniform red color | 85.0 | 85.3 |
| TG | 1 | relatively firm, dark black with brownish tones, still lustrous, reasonably uniform, and relatively clean | 76.0 | with floral | 74.0 | bright orange-red | 78.0 | smooth and mellow, with a hint of floral | 73.5 | still uniform red color | 75.0 | 74.9 |
|  | 2 | still tight and compact, dark brown and lustrous, reasonably uniform, and relatively clean | 74.5 | with floral | 75.0 | still bright orange-red | 76.0 | smooth and mellow, with a hint of floral | 74.0 | still uniform red color | 75.5 | 74.7 |
|  | 3 | relatively tight and compact, dark black and lustrous, fairly uniform, and relatively clean | 75.0 | with floral | 77.0 | still bright orange-red | 75.0 | relatively smooth and mellow, with a hint of floral and fruity | 78.0 | still uniform red color | 74.0 | 76.3 |


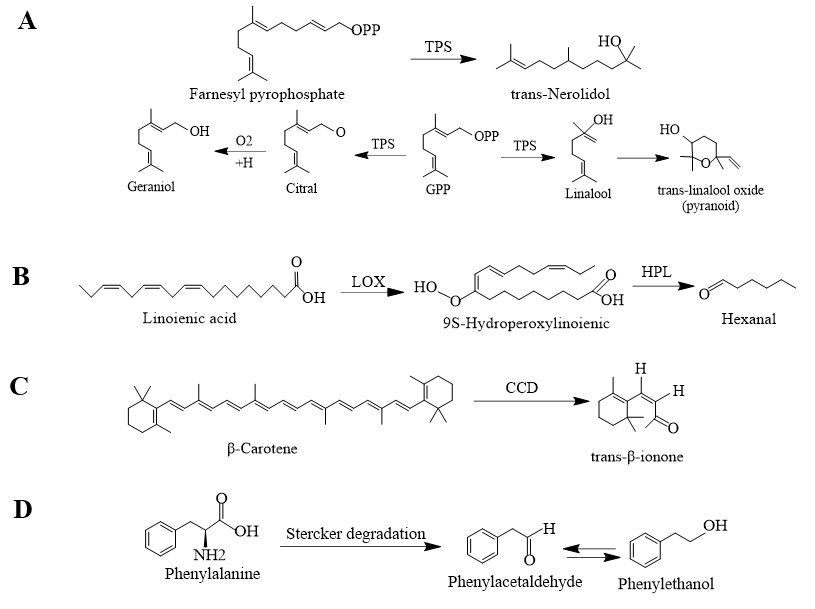


**Fig. S1:** Pathways for the formation of 7 characteristic substances of key aromas. (A) Formation pathways of volatile terpenoids (trans-nerolidol, geraniol, citral, linalool); (B) Fatty acid derivative (hexanal) formation pathway; (C) Pathway of carotenoid derivatives ((E)-β-ionone or also known as *trans*-β-ionone); (D) Formation pathways of volatile phenylpropanoids/benzene ring compounds (phenylacetaldehyde)
